# Chemical Characterization and Antimicrobial Activity of Essential Oils and Nanoemulsions of *Eugenia uniflora* and *Psidium guajava*

**DOI:** 10.3390/antibiotics14010093

**Published:** 2025-01-14

**Authors:** Rebeca Dias dos Santos, Breno Noronha Matos, Daniel Oliveira Freire, Franklyn Santos da Silva, Bruno Alcântara do Prado, Karolina Oliveira Gomes, Marta Oliveira de Araújo, Carla Azevedo Bilac, Letícia Fernandes Silva Rodrigues, Izabel Cristina Rodrigues da Silva, Lívia Cristina Lira de Sá Barreto, Claudio Augusto Gomes da Camara, Marcilio Martins de Moraes, Guilherme Martins Gelfuso, Daniela Castilho Orsi

**Affiliations:** 1 Laboratory of Quality Control, University of Brasília (UNB/FCE), Centro Metropolitano, Conjunto A, Lote 01, Ceilândia, Brasília 72220-900, DF, Brazil; rebecadias123@gmail.com (R.D.d.S.); daniel.microbiologia@gmail.com (D.O.F.); franklyn.silva@aluno.unb.br (F.S.d.S.); prado.bruno@aluno.unb.br (B.A.d.P.); gomes.karolina@aluno.unb.br (K.O.G.); oa.martaaraujo@gmail.com (M.O.d.A.); carlabilac@gmail.com (C.A.B.); rodriguesleticiafs@gmail.com (L.F.S.R.); belbiomedica@gmail.com (I.C.R.d.S.); liviabarreto@unb.br (L.C.L.d.S.B.); 2 Laboratory of Food, Drugs, and Cosmetics, University of Brasília (UNB/FS), Brasília 70910-900, DF, Brazil; brenomatos15@hotmail.com (B.N.M.); gmgelfuso@unb.br (G.M.G.); 3 Department of Chemistry, Federal Rural University of Pernambuco, Recife 50740-560, PE, Brazil; claudio.camara@ufrpe.br (C.A.G.d.C.); marcilio.moraes@ufrpe.br (M.M.d.M.)

**Keywords:** pitanga essential oil, guajava essential oil, nanoemulsion, antifungal activity, antibacterial activity

## Abstract

**Background/Objectives:** This study aimed to develop gel nanoemulsions (NEs) of Brazilian essential oils (EOs) from *Eugenia uniflora* and *Psidium guajava*, as well as to perform chemical characterization and investigate the antimicrobial activity of the EOs and NEs. **Results/Conclusions**: The main chemical compounds of *E. uniflora* EO were curzerene (34.80%) and germacrene B (11.92%), while those of *P. guajava* EO were β-caryophyllene (25.92%), β-selinene (22.64%), and γ-selinene (19.13%). The NEs of *E. uniflora* and *P. guajava* had droplet sizes of 105.30 and 99.50 nm and polydispersity index (PDI) values of 0.32 and 0.43, respectively. The NEs remained stable for 30 days of storage at 25 °C, with droplet sizes of 104.7 and 103.8 nm, PDI values below 0.50, and no phase separation. The NE of *E. uniflora* exhibited inhibition zones ranging from 8.41 to 15.13 mm against the Gram-positive bacterium *Staphylococcus aureus* and the Gram-negative bacteria *Pseudomonas aeruginosa*, *Klebsiella pneumoniae*, and *Acinetobacter baumannii*. Additionally, the NE of *E. uniflora* showed the largest inhibition zones against *Candida albicans* (20.97 mm) and *Candida krusei* (15.20 mm), along with low minimum inhibitory concentration (MIC) values (0.54–1.22 mg/mL) and minimal bactericidal concentration (MBC) values (4.84–11.02 mg/mL) against these pathogenic yeasts. The NE of *P. guajava* demonstrated low MIC (1.26 mg/mL) and MBC (11.35 mg/mL) values for *C. krusei*. The time–growth inhibition assay also suggests the effectiveness of the NE against the tested pathogens *S. aureus* and *E. coli*, highlighting its potential as a novel alternative therapeutic agent.

## 1. Introduction

*Eugenia uniflora* L., popularly known as pitanga or Brazilian cherry, belongs to the Myrtaceae family and is a fruit tree native to Brazil. Traditional communities use an infusion of the leaves and bark of *E. uniflora* to treat diarrhea, fever, and inflammation [1,2,3]. *Psidium guajava*, commonly known as guava, is a fruit tree native to South and Central America and belongs to the Myrtaceae family. In traditional medicine, guava leaves are used as laxatives and to treat wounds, gastrointestinal problems, and diabetes [4,5,6].

Essential oils of both *E. uniflora* and *P. guajava* are extracted from the leaves and contain a complex mixture of compounds, including monoterpenes, sesquiterpenes, and phenolic compounds (flavonoids and phenols). They exhibit biological properties such as antimicrobial (antibacterial and antifungal), antioxidant, larvicidal, and anti-inflammatory effects [1,2,3,4,5].

Antimicrobial resistance is a global problem, creating an increasing need for new therapeutic alternatives to combat resistant microorganisms, as well as for the discovery of new antimicrobial drugs with novel chemical structures and mechanisms of action [7,8]. The Gram-positive bacterium *Staphylococcus aureus*; the Gram-negative bacteria *Pseudomonas aeruginosa*, *Klebsiella pneumoniae*, *Acinetobacter baumannii*, and *Escherichia coli*; and the yeasts *Candida albicans* and *Candida krusei* are important microorganisms that cause a wide variety of diseases in humans and currently exhibit increasing resistance to common antimicrobial agents [9,10,11,12,13,14,15,16].

Given this scenario, many studies are exploring the potential of natural products as possible therapeutic alternatives to help treat infections caused by resistant microorganisms. Essential oils contain a complex mixture of chemical compounds in their composition, which exhibit antimicrobial activity [17,18]. One of the advantages of specifically using the essential oils of *E. uniflora* and *P. guajava* as antimicrobials, compared to other essential oils, is the exploration of the biodiversity of the Brazilian flora, since these essential oils are less studied in the literature [19].

Despite their numerous advantages, essential oils face limitations as therapeutic agents in their free form, with one of the main limitations being their poor solubility in water, which affects their bioavailability and efficacy. Additionally, essential oils are highly volatile and can degrade over time, reducing their effectiveness [18]. An alternative to improve the stability and ensure the efficacy of essential oils is the production of nanoemulsions. Incorporating essential oils into nanoemulsions protects the active ingredient from degradation, increases solubility, and enhances biological activity. These systems create nanoscale droplets, significantly increasing the surface area of essential oils. The large surface area provided by the nanometric size increases solubility, resulting in greater bioavailability of poorly water-soluble compounds. Thus, nanoemulsions can encapsulate, protect, and promote the controlled release of lipophilic bioactive molecules [20].

Although some studies have reported the antimicrobial activities of Brazilian essential oils from *E. uniflora* and *P. guajava* [2,3,4,5], the formulation of nanoemulsions with these essential oils is still scarce in the literature [21], and no studies were found regarding the nanoemulsion of the essential oil from *E. uniflora*. Nanoemulsions have been identified as a promising means for the delivery of antimicrobial agents due to their ability to enhance the solubility, stability, and bioavailability of essential oils; their potential for organ and cellular targeting; their ability to penetrate biofilms; and their potential to overcome antimicrobial resistance [22]. Thus, gel-based nanoemulsions of *E. uniflora* and *P. guajava* could represent an innovative approach for the development of dressings to treat infected wounds and burns.

In the development of nanoemulsions, it is fundamentally important to study the correct formulation that results in nanoemulsions with long-term stability and scalability [23]. Therefore, this study aimed to develop gel nanoemulsions of the essential oils from *E. uniflora* and *P. guajava*, as well as to perform a chemical characterization and study the antimicrobial activity of the essential oils and nanoemulsions.

## 2. Results and Discussion

### 2.1. Chemical Characterization of E. uniflora and P. guajava Essential Oils

Table 1 and Table 2 show the chemical composition of the essential oils of *E. uniflora* and *P. guajava*. The analysis of the chemical composition of the *E. uniflora* essential oil allowed the identification of 31 volatile compounds, representing 98.60% of the total oil, while the *P. guajava* essential oil revealed the identification of 21 volatile compounds, representing 98.10% of the total oil.

The major compounds classes in the essential oil of *E. uniflora* were sesquiterpenes (92.69%), with a predominance of sesquiterpene hydrocarbons (51.96%), oxygenated sesquiterpenes (40.73%), and monoterpenes (5.91%). The chemical constituents present in the largest amounts were the oxygenated sesquiterpene curzerene (34.80) and the sesquiterpene hydrocarbon germacrene B (11.92%). Other constituents were also identified in significant quantities, such as the sesquiterpene hydrocarbons β-cubebene (6.79%), aristolene (5.73%), β-caryophyllene (5.16%), β-elemene (4.74%), and calamenene (4.62%) and the oxygenated sesquiterpene germacrone (3.99%).

De Jesus et al. [3] analyzed an essential oil of *E. uniflora* from the northern region of Brazil (Belém City, Pará State) and obtained similar results to our study, where the major compound was curzerene (33.40%). Da Costa et al. [1] evaluated the influence of seasonality on the chemical composition of *E. uniflora* essential oil from the northern region of Brazil (Belém City, Pará State) and observed that curzerene was the main constituent; its percentage showed no significant difference between the dry season (42.70%) and the rainy season (40.80%). Other constituents, such as the oxygenated sesquiterpenes germacrone (0.20–10.50%), globulol (1.50–7.40%), and spathulenol (0.50–7.00%) and the sesquiterpene hydrocarbons germacrene B (0.10–7.50%) and β-elemene (1.80–5.80%), were also identified in the *E. uniflora* essential oil. da Silva et al. [2] identified selina-1,3,7(11)-trien-8-one (57.55%) and oxido-selina-1,3,7(11)-trien-8-one (21.18%) as the major compounds in *E. uniflora* essential oil from the northeastern region of Brazil (Recife City, Pernambuco State).

Figueiredo et al. [24] reported that the chemical composition of *E. uniflora* essential oil has significant variability with different chemotypes but, in general, is rich in cyclic oxygenated sesquiterpenes with a germacrane-type skeleton, such as curzerene, selina-1,3,7(11)-trien-8-one, and selina-1,3,7(11)-trien-8-one epoxide, germacrone, and in association with other cyclic sesquiterpene hydrocarbons. The results of our study indicated that the chemical profile of *E. uniflora* essential oil was similar to that described by Figueiredo et al. [24] for chemotype III, which was represented by curzerene (50.6%), germacrene B (5.20%), and germacrone (4.50%).

The essential oil of *E. uniflora* contained 34.80% curzerene in its composition. Other than the essential oil of *E. uniflora*, only a few essential oils contain significant amounts of curzerene in their composition. Doodman et al. [25] reported 23.96 to 47.04% curzerene in the essential oil of *Smyrnium olusatrum*, an aromatic plant native to the Iran–Turkey region. The *Curcuma* genus also contains curzerene in significant quantities in the essential oils of its species (*C. aeruginosa* presented 4.70%, and *C. zedoaria* presented 6.20% of curzerene) [26].

Although there are few studies in the literature on the antimicrobial activity of curzerene, essential oils with curzerene as a major component exhibit inhibitory effects against various microorganisms. The antimicrobial activity of curzerene can be attributed to its structural characteristics, which allow it to interact with microbial membranes in a similar manner to other sesquiterpenes. This interaction disrupts membrane integrity, leading to cell death [27].

The major compounds classes in the essential oil of *P. guajava* were sesquiterpenes (92.16%), with a predominance of sesquiterpene hydrocarbons (81.30%), oxygenated sesquiterpenes (10.86%), and monoterpenes (5.94%). The chemical constituents with the highest amounts were the sesquiterpene hydrocarbons β-caryophyllene (25.92%), β-selinene (22.64%), and γ-selinene (19.13%). Other constituents were also identified in significant quantities, such as the oxygenated sesquiterpenes caryophyllene oxide (5.61%) and selin-6-en-4-α-ol (3.54%), the monoterpene eucalyptol (4.58%), and the sesquiterpene humulene (3.95%) (Table 2).

Dos Santos et al. [28] obtained similar results when analyzing the essential oil of *P. guajava* from the southeastern region of Brazil (Rio de Janeiro City, Rio de Janeiro State), identifying 20 compounds, of which 85.52% were sesquiterpene hydrocarbons. The major compounds were represented by α-humulene (33.20%), (E)-caryophyllene oxide (25.83%), α-selinene (12.08%), and β-selinene (11.71%). De Souza et al. [5] evaluated the essential oil of *P. guajava* from the southern region of Brazil (Canelinha City, Santa Catarina State) and identified β-selinene (13.83%), α-humulene (10.90%), and β-caryophyllene (7.61%) as the main compounds. Souza et al. [29] reported that essential oils of *P. guajava* from 22 different genotypes cultivated in the southeastern region of Brazil (Mimoso do Sul and Linhares Cities, Espírito Santo State) showed a predominance of trans-caryophyllene (14.2–16.0%) and α-humulene (7.1–11.4%). In five genotypes, there was also a prevalence of caryophyllene oxide (9.5–11.5%), β-selinene (6.8–8.0%), and α-selinene (6.1–7.6%).

The essential oil of *P. guajava* contained 41.77% selinene and 25.92% β-caryophyllene in its composition. Sarma et al. [30] reported similar results for the essential oil of *Lantana camara* leaves, which had β-caryophyllene (24.96%) and δ-selinene (17.46%) as major compounds. Some other essential oils have also been reported in the literature as having selinene as a major component, such as *Artemisia annua* (β-selinene 20.05–46.29%) [31] and *Callicarpa macrophylla* (β-selinene 37.51–57.01%) [32].

The sesquiterpene β-caryophyllene is studied in the literature for its antimicrobial activities. Dahham et al. [33] isolated β-caryophyllene from the essential oil of *Aquilaria crassna* and observed that the compound exhibited high antimicrobial activity, particularly against Gram-positive bacteria. Woo et al. [34] evaluated the antimicrobial activity of β-caryophyllene against the bacterium *Helicobacter pylori* and reported an MIC value of 1.0 mg/mL. The authors concluded that β-caryophyllene disrupts bacterial replication by downregulating the genes *dnaE*, *dnaN*, *holB*, and *gyrA*, which are expressed by *H. pylori*.

In the present study, *E. uniflora* essential oil showed higher antioxidant activity and phenolic compound content than *P. guajava* essential oil (Table 3). The antioxidant activity of essential oils can be attributed to their major chemical compounds, acting either individually or in synergy. Both terpenes and phenolic compounds are known for their antioxidant activity [35]. The antioxidant properties of essential oils can support their antimicrobial efficacy, as they protect the oils from oxidation during storage, preserving their bioactive compounds and ensuring consistent antimicrobial effectiveness over time [33,34].

Oxygenated sesquiterpenes have higher antioxidant activity than sesquiterpene hydrocarbons, as the free radical scavenging activities are directly correlated with the oxygenation concentration of their constituents due to the increase in free electrons [36]. Thus, the higher oxygenated sesquiterpene content in *E. uniflora* essential oil (40.73%) may contribute to its enhanced antioxidant activity compared to *P. guajava* essential oil (10.86%), which has a lower concentration of these compounds.

*E. uniflora* essential oil presented antioxidant activity of 853.62 μmol TE/mL (ABTS) and 183.71 μmol TE/mL (DPPH). Other studies have also reported antioxidant activity in Brazilian essential oils from *E. uniflora*. Da Costa et al. [37] reported that two different samples of *E. uniflora* essential oil from leaves collected in the northern region of Brazil showed DPPH radical inhibition of 45.1% (228.3 mg TE/g) and 42.8% (217.0 mg TE/g). Additionally, Da Costa et al. [1] reported that *E. uniflora* essential oils, obtained from twelve months (January to December) of plant leaf sample collection in the Brazilian Amazon region, demonstrated DPPH antioxidant activity ranging from 186.9 to 436.3 mg TE/g.

*P. guajava* essential oil presented antioxidant activity of 204.19 μmol TE/mL (ABTS) and 51.30 μmol TE/mL (DPPH). Jeronimo et al. [38] reported that *P. guajava* essential oil from the northern region of Brazil presented a total antioxidant capacity of 195.7 mg TE/mL (DPPH). de Souza et al. [5] reported that the essential oil of *P. guajava* from the south region of Brazil showed 4.54 and 8.94 μmol TE/mL for ABTS and DPPH, respectively.

*E. uniflora* and *P. guajava* essential oils presented values of total phenolic content of 1.30 and 1.10 mg GAE/mL, respectively. Omurtag Özgen et al. [39] reported a similar value of total phenolic content of 1.22 mg GAE/g for *Lavandula angustifolia* Mill. essential oil from Turkey. Mechergui et al. [40] obtained values of total phenolic content of *Origanum vulgare* essential oils from North Africa varying from 4.10–17.70 mg GAE/g, and El-Demerdash [41] reported that the essential oil of *Rosmarinus officinalis* from Egypt contained a concentration of phenolic content of 0.04 mg GAE/mL.

### 2.2. Characterization and Stability of Nanoemulsions of E. uniflora and P. guajava

The nanoemulsions of *E. uniflora* and *P. guajava* had droplet sizes of 105.30 and 99.50 nm, respectively (Table 4). According to the literature, nanoemulsions are colloidal dispersions composed of nanoscale droplets, with sizes ranging from 20 to 200 nm. The smaller droplet size in nanoemulsions helps suppress coalescence and precipitation of the droplets, and this contributes to minimizing the degradation of the nanoemulsion and increasing its stability [42,43].

The results of the present study are similar to the droplet sizes reported for nanoemulsions of other essential oils. A nanoemulsion of *Thymus vulgaris* essential oil showed spherical droplets with a diameter of 127.6 nm [44], while a cinnamon essential oil nanoemulsion resulted in droplets with an average size of 162.1 nm [45]. Additionally, it was reported that the hydrodynamic diameters of the nanoemulsions formulated with *Carlina acaulis* essential oil ranged between 110 and 140 nm [46].

The polydispersity index obtained was 0.32 and 0.43 for the nanoemulsions of *E. uniflora* and *P. guajava*, respectively. The polydispersity index (PDI) measures the variation in particle sizes within a sample. It ranges from 0.0, which indicates a sample with uniform particle sizes, to 1.0, which reflects a sample with a wide variation in particle sizes. A PDI below 0.08 indicates a monodisperse distribution, while values above 0.70 suggest a broader droplet size distribution. A PDI between 0.08 and 0.70 is considered an acceptable size distribution in nanoemulsions. Thus, the PDI is an important indicator of homogeneity and is generally used to determine the stability of nanoemulsions [47,48].

Dakhlaoui et al. [49] reported similar results, in which nanoemulsions of *Eucalyptus cladocalyx* essential oil showed a PDI of 0.49. Additionally, Soulaimani et al. [50] obtained PDI variations between 0.20 and 0.44 for nanoemulsions obtained from a mixture of essential oils of *Lavandula maroccana*, *T. vulgaris*, and *Ammodaucus leucotrichus*.

The zeta potential obtained was −9.29 and −7.34 mV for the nanoemulsions of *E. uniflora* and *P. guajava*, respectively. The zeta potential is used to measure the surface charge of particles in a liquid suspension, determined by the speed at which charged particles move toward the electrode when exposed to an external electric field, with values ranging from +100 to −100 mV. The zeta potential is influenced by physicochemical properties, the presence of electrolytes, and the adsorption capacity of the nanoparticles [51]. Non-ionic surfactants, such as Tween, Cremophor^®^ EL, and Plurol^®^ Oleique, create strong repulsive steric forces or osmotic repulsive forces by forming a barrier around the oil droplet, thus stabilizing the emulsion and preventing droplet aggregation [52].

Morteza-Semnani et al. [48] observed zeta potential values from −0.50 to −6.90 mV for nanoemulgel formulations containing cumin essential oil. Moazeni et al. [44] obtained a zeta potential value of −9.82 mV for *T. vulgaris* essential oil nanoemulsion.

In this study, nanoemulsions of *E. uniflora* and *P. guajava* resulted in pH values of 5.75 and 5.41, respectively. Human skin has a pH ranging between 4.20 and 5.60; therefore, solutions with a pH closer to that of the skin favor nanoparticles penetration, as they can reduce electrostatic forces, thereby facilitating skin permeation [53].

**Figure 1:**
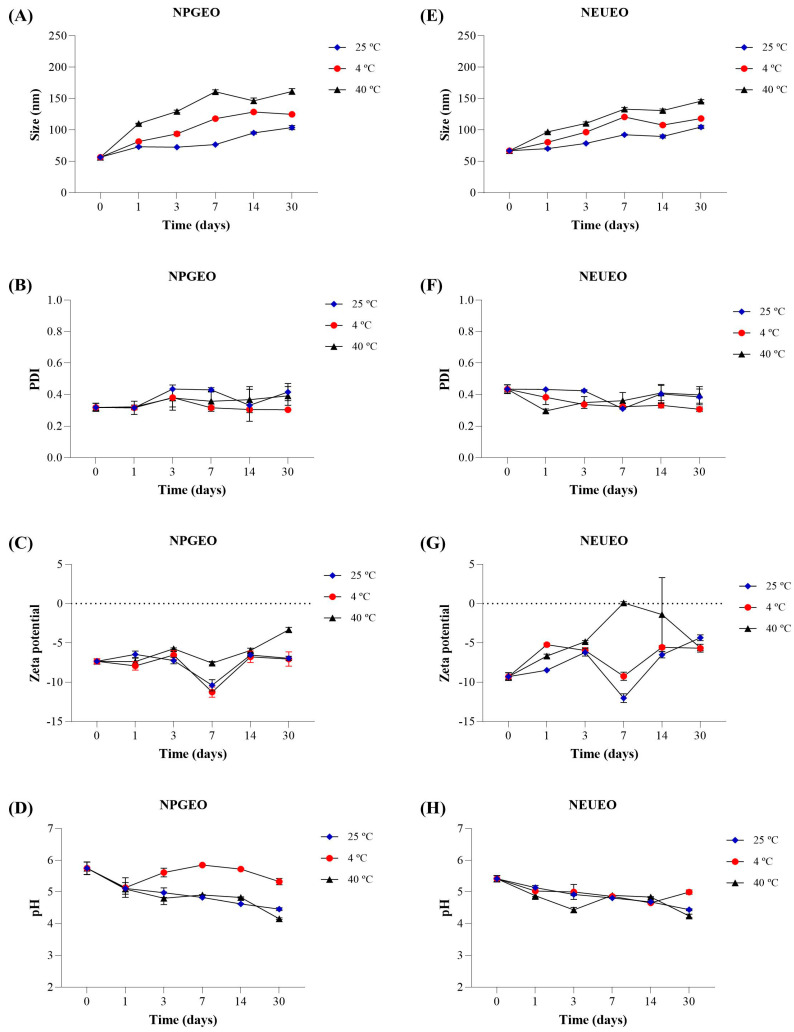
Effects of temperature (4, 25, and 40 °C) and storage time (0, 1, 3, 7, 14, and 30 days) on stability of nanoemulsions of *P. guajava* and *E. uniflora*. (**A**–**D**) = stability of nanoemulsion of *P. guajava* essential oil (NPGEO), (**A**)-nanoemulsion size (nm), (**B**)-polydispersity index (PDI), (**C**)-zeta potential, and (**D**)-pH. (**E**–**H**) = stability of nanoemulsion of *E. uniflora* essential oil (NEUEO), (**E**)-nanoemulsion size (nm), (**F**)-polydispersity index (PDI), (**G**)-zeta potential, and (**H**)-pH. Results are expressed as mean and standard deviation.

The nanoemulsions of *E. uniflora* and *P. guajava* were investigated for the effects of temperature (4, 25, and 40 °C) and storage time (0, 1, 3, 7, 14, and 30 days) on stability. The changes in droplet size, PDI, zeta potential, and pH values are presented in Figure 1. The droplet size at all storage temperatures increased over time; however, no phase separation was observed, and the droplet sizes of the nanoemulsions remained below 200 nm for 30 days. At a temperature of 25 °C, a smaller change in droplet size was observed, ranging from 73.13 to 103.80 nm for the *P. guajava* nanoemulsion and from 70.26 to 104.7 nm for the *E. uniflora* nanoemulsion. On the other hand, storage at 40 °C showed greater variation in the droplet size of the nanoemulsions over time.

The low PDI values of the *E. uniflora* and *P. guajava* nanoemulsions, which remained below 0.50 at all storage temperatures, indicated the size homogeneity of the formulations. The low PDI values of the *E. uniflora* and *P. guajava* nanoemulsions, which remained below 0.50 at all storage temperatures, indicated the size homogeneity of the formulations. Nanoemulsions with low PDI values (<0.50) exhibit a more homogeneous particle distribution, which is crucial for ensuring their stability, as they are less prone to phase separation. A PDI < 0.50 directly impacts the antibacterial activity of nanoemulsions because uniform particles penetrate microbial membranes more easily [54,55]. Additionally, the PDI enhances the diffusion of the nanoemulsion into bacterial cell walls, as smaller and more uniformly sized droplets increase the surface-area-to-volume ratio of the nanoemulsion [56].

The zeta potential exhibited a negative charge, ranging from −7.34 to −6.97 mV at 25 °C for the *P. guajava* nanoemulsion and from −8.48 to −6.51 mV at 25 °C for the *E. uniflora* nanoemulsion. At 4 °C, the zeta potential showed similar variation to that at 25 °C. However, at 40 °C, there was a greater variation in the zeta potential of the nanoemulsions.

The electrical properties (zeta potential) have a significant impact on nanoemulsion stability and are influenced by the surfactants used in formulation. While non-ionic surfactants, such as those used in our study (Cremophor^®^ EL and Plurol^®^ Oleique), are stabilized by dipole interactions and hydrogen bonding with the water hydration layer, as well as by repulsive forces due to steric hindrance, ionic surfactants are additionally stabilized by electrostatic interactions. However, non-ionic surfactants are the first choice in nanoemulsion formulations because, compared to ionic surfactants, they exhibit a safer toxicological profile [57].

According to Somala et al. [58], temperature influences the stability of emulsions, affecting the physical properties of oil, water, and surfactant. The results of the present study indicated a possible loss of stability in nanoemulsions stored at 40 °C for a period beyond 30 days, while temperatures of 25 and 4 °C showed better stability for the nanoemulsions over 30 days of storage.

Somala et al. [58] reported similar results for nanoemulsions containing citronella (*Cymbopogon nardus*) essential oil. At a temperature of 4 °C, the mean droplet size remained below 200 nm; however, nanoemulsions stored at 45 °C exhibited a larger droplet size, increasing from 79 to 218 nm after 14 days. Yin et al. [59] reported good stability of a nanoemulsion containing mangosteen extract and kojic acid for 42 days at a temperature of 25 °C, with a droplet size of 162.9 nm, a PDI of 0.39, and no phase separation.

The stability of nanoemulsions under storage conditions is crucial for practical applications, such as in topical formulations or food systems [60,61]. Additional studies are necessary to evaluate the stability of nanoemulsions when exposed to environmental factors such as light and humidity.

### 2.3. Antimicrobial Activity of Essential Oils and Nanoemulsions of P. guajava and E. uniflora

The nanoemulsion of *E. uniflora* showed the highest antimicrobial activity of all the tested compounds according to the agar-well diffusion method (Table 5), with inhibition zones ranging from 8.41 to 20.97 mm against all tested microorganisms except for *E. coli*, which showed no inhibition zone for any of the tested compounds. The largest inhibition zones were observed against the yeasts *C. albicans* (20.97 mm) and *C. krusei* (15.20 mm).

The antimicrobial properties of essential oils can be primarily attributed to their ability to interact with biological membranes, affecting membrane potential and, consequently, permeability, nutrient transport, and ion exchange. The small droplets of nanoemulsions enhance the interactions between essential oils and the bacterial cell wall, thereby amplifying their antimicrobial efficacy. Thus, cell rupture is one of the main mechanisms by which nanoemulsions exert their antimicrobial effects. This occurs due to the small droplet size of the nanoemulsions, which enables rapid diffusion of their compounds into the cell wall, causing cell lysis [62,63]. When essential oils are present in the nanoemulsion composition, they induce morphological changes in cells, such as deformation and leakage of intracellular contents, resulting in cell death [64,65]. Additionally, protein denaturation and alterations in surface hydrophobicity are critical factors in the antimicrobial action of nanoemulsions. Encapsulated essential oils in nanoemulsions lead to protein denaturation in bacterial cells, contributing to the loss of structural integrity and function. This denaturation process is often accompanied by the release of cytoplasmic materials, which is indicative of cell death [63].

Similarly, Jawaid et al. [66] reported that citronella essential oil nanoemulsion showed better zones of inhibition against *S. aureus* and *C. albicans* than citronella essential oil. Hassanshahian et al. [67] also observed smaller inhibition zones against the bacteria *S. aureus*, *P. aeruginosa*, and *A. baumannii* for the free essential oil of *Alhagi maurorum* leaves than for the nanoemulsion.

The nanoemulsion of *P. guajava* showed lower antimicrobial activity using the agar-well diffusion method than the nanoemulsion of *E. uniflora*, with inhibition zones ranging from 6.06 to 10.05 mm against the microorganisms *K. pneumoniae*, *P. aeruginosa*, *A. baumannii*, and *S. aureus* and no antimicrobial activity against the yeasts *C. albicans* and *C. krusei*.

The essential oil of *E. uniflora* presented inhibition zones ranging from 6.67 to 14.30 mm against the Gram-positive bacterium *S. aureus* and the yeasts *C. albicans* and *C. krusei*, with no antimicrobial activity observed against the tested Gram-negative bacteria. Similarly, Fidelis et al. [68] reported that the essential oil from *E. uniflora* showed inhibition zones for Gram-positive pathogens such as *S. aureus*, *Listeria monocytogenes*, *Bacillus subtilis*, and *Streptococcus faecalis*, while no antimicrobial activity was observed against Gram-negative pathogens. Mohamed et al. [69] investigated the antimicrobial activities of the essential oil from *E. uniflora* leaves cultivated in Egypt and found an inhibition zone of 12 mm for the oil diluted to 20% for *C. albicans*. Ferreira et al. [70] analyzed the essential oil from *Eugenia florida* leaves collected in the northern region of Brazil (Magalhães Barata City, Pará State) and obtained inhibition zones ranging from 6 to 8 mm for *C. albicans*, *C. tropicalis*, *C. famata*, *C. krusei*, and *C. auris*.

In this study, the essential oil of *P. guajava* only showed activity against *S. aureus*, with inhibition zones ranging from 9.10 to 10.93 mm. *P. guajava* essential oils from China showed inhibition halos against different strains of *S. aureus* ranging from 9.00 to 18.60 mm [71]. *P. guajava* essential oil from the southern region of Brazil (Canelinha City, Santa Catarina State) exhibited an inhibition halo against *S. aureus* of 16.07 mm [5].

It is interesting to note that the essential oils diluted to 15% produced larger inhibition zones than the pure essential oils, indicating a diffusion challenge for lipidic compounds in agar. The nanoemulsions become more soluble in an aqueous medium, which likely contributed to enhancing antimicrobial activity in the agar-well diffusion methodology. Although the agar diffusion methodology is widely used to evaluate the antimicrobial activity of essential oils, it is recommended that this method be complemented by the determination of minimum inhibitory concentration (MIC) and minimum bactericidal concentration (MBC), which makes it possible to establish the minimum doses of the compound that have a bacteriostatic and bactericidal effect, respectively [72].

Table 6 presents the MIC and MBC values of *E. uniflora* and *P. guajava* essential oils and their nanoemulsions. The nanoemulsion of *E. uniflora* showed lower MIC (0.54–1.22 mg/mL) and MBC (4.84–11.02 mg/mL) values against the yeasts *C. albicans* and *C. krusei* than the essential oil of *E. uniflora*. The nanoemulsion of *P. guajava* showed lower MIC values (1.26 mg/mL) and MBC values (11.35 mg/mL) against *C. krusei* than the essential oil of *P. guajava*, indicating potential for using these nanoemulsions as antifungal agents against these two important human pathogens. Similarly, Jawaid et al. [66] reported that the nanoemulsion of citronella essential oil showed a lower MIC value (0.13 mg/mL) against *C. albicans* than citronella essential oil (0.25 mg/mL). Moazeni et al. [44] observed that the antifungal activity of a thyme essential oil-based nanoemulsion was higher than that of thyme essential oil against *C. albicans*, *C. glabrata*, and *C. parapsilosis*.

For the Gram-negative bacteria *K. pneumoniae* and *A. baumannii*, the essential oil of *E. uniflora* showed the lowest MIC (0.25–1.39 mg/mL) and MBC (2.34–12.49 mg/mL) values among the tested compounds. For the Gram-positive bacterium *S. aureus*, the essential oil of *E. uniflora* presented an MIC value of 1.95 mg/mL and an MBC value of 17.53 mg/mL. Antonelo et al. [73] reported that the essential oil of *E. uniflora* from the southeast region of Brazil (City of Ibiúna, State of São Paulo) showed similar antimicrobial activity when tested against *S. aureus* (MIC of 3.13 mg/mL and MBC of 12.50 mg/mL). Obuotor et al. [74] analyzed the essential oil of *E. uniflora* obtained from leaves collected in Ota, Nigeria, Africa, and obtained the following values: against *S. aureus*, an MIC of 25.00 mg/mL and an MBC of 50.00 mg/mL; against *K. pneumoniae*, an MIC and an MBC of 50.00 mg/mL; and against *P. aeruginosa*, an MIC of 50.00 mg/mL and an MBC of 100.00 mg/mL.

For the Gram-positive bacterium *S. aureus* and the Gram-negative bacterium *E. coli*, the essential oil of *P. guajava* showed the lowest MIC (0.42–1.13 mg/mL) and MBC (3.86–10.16 mg/mL) values among the tested compounds. Hanif et al. [75] reported similar results with the essential oils extracted from the leaves of two *P. guajava* cultivars (white and pink fruits) from the local fields of Faisalabad, Punjab, Pakistan. *P. guajava* essential oils exhibited MIC values of 2.50–3.13 against *S. aureus* and MIC values of 8.50–12.5 mg/mL against *E. coli*.

Thus, in the present study, for the tested bacteria (*S. aureus, P. aeruginosa, K. pneumoniae, A. baumannii,* and *E. coli*), the nanoemulsions exhibited higher MIC and MBC values than the essential oils. Nevertheless, the nanoemulsions exhibited MIC values (3.38–20.08 mg/mL against *E. uniflora* and 3.52–23.50 mg/mL against *P. guajava*) comparable to those reported in the literature. Hassanshahian et al. [67] observed that an *A. maurorum* essential oil nanoemulsion had MIC values of 6.25 mg/mL against *S. aureus*, 1.75 against *E. coli*, 12.5 mg/mL against *A. baumannii* and *P. aeruginosa*, and 25.0 mg/mL for *K. pneumoniae*. Shehabeldine et al. [76] reported that a clove essential oil nanoemulsion had antimicrobial activity values of 1.25 mg/mL against *S. aureus*, 2.50 mg/mL against *E. coli*, and 10 mg/mL against *Klebsiella oxytoca*.

Studies in the literature have reported the synergistic effect of nanoemulsions of essential oils and synthetic antimicrobials. Synergistic interactions enhance antimicrobial and antioxidant activity by utilizing the efficiencies of the combined agents in the best possible manner and thereby result in a severalfold reduction in the required doses of the combined agents. Sharma et al. [77] reported that the synergistic interactions in binary mixtures of essential oils from *Callistemon lanceolatus*, *Ocimum gratissimum*, *Cymbopogon winterianus*, *Cymbopogon flexuosus*, *Mentha longifolia*, and *Vitex negundo* with synthetic compounds (chloramphenicol and ampicillin) enhanced the antimicrobial activity of the combinations in vitro. The resulting reduction in the effective doses of the synthetic compounds may lower the adverse effects, high costs, toxicity, and multi-drug resistance associated with their large doses when used alone.

**Figure 2:**
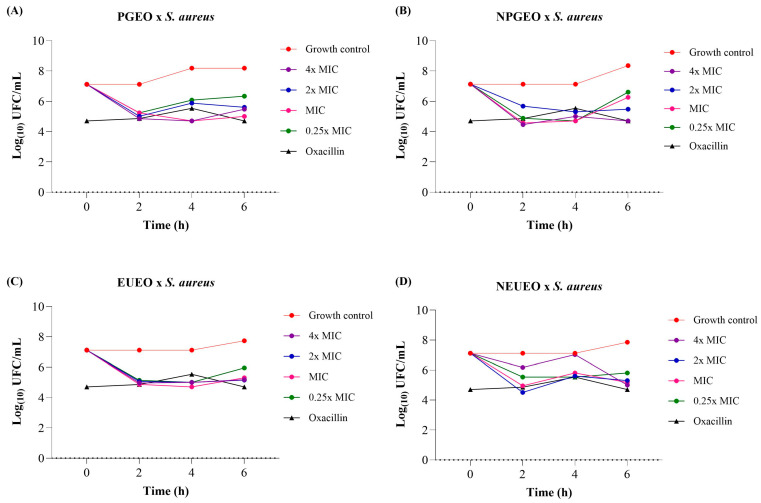
Time–growth inhibition assay of *S. aureus* for different concentrations of (**A**) *P. guajava* essential oil (PGEO), (**B**) *P. guajava* nanoemulsion (NPGEO), (**C**) *E. uniflora* essential oil (EUEO), and (**D**) *E. uniflora* nanoemulsion (NEUEO). Based on the MIC determined for the essential oil and nanoemulsion, the tested concentrations were defined as 4 × MIC, 2 × MIC, MIC, and 0.25 × MIC. The control growth corresponds to positive microbial growth, and the MIC of oxacillin was used as a growth inhibition control.

Time–growth inhibition assays of *E. uniflora* and *P. guajava* essential oils and nanoemulsions against *S. aureus* were evaluated (Figure 2). This test provides information about the dynamic interaction between the antimicrobial agent and the microbial strain. Additionally, the test reveals whether the antimicrobial effect is time-dependent and/or concentration-dependent [72].

The bacterium *S. aureus* presented an initial inoculum size at time 0 of 7.12–7.13 log CFU/g. After 6 h of incubation, at all tested MIC concentrations, the *P. guajava* essential oil showed a count of 5.00–6.34 log CFU/g (Figure 2A), the *P. guajava* nanoemulsion showed a count of 4.70–6.60 log CFU/g (Figure 2B), the *E. uniflora* essential oil showed a count of 5.18–5.95 log CFU/g (Figure 2C), and the *E. uniflora* nanoemulsion showed a count of 5.00–5.81 log CFU/g (Figure 2D), indicating a reduction in the original inoculum size of the bacterium *S. aureus*.

At 6 h, the positive microbial growth control in tests of *P. guajava* essential oil and its nanoemulsion was 8.19–8.35 log CFU/g, and for *E. uniflora* essential oil and its nanoemulsion, the range was 7.74–7.85 log CFU/g. All tested compounds demonstrated a reduction of at least 2.0 log cycles in the *S. aureus* count compared to the positive microbial growth control.

Thus, in the time–growth inhibition assay with the essential oils and nanoemulsions of *E. uniflora* and *P. guajava*, the bacteriostatic activity (MIC) values of these compounds were used. The results against *S. aureus* demonstrated bacteriostatic activity after 6 h of exposure to the tested compounds, showing antimicrobial activity equivalent to that of the antibiotic oxacillin, which presented values ranging from 4.70 to 6.19 log CFU/g in 6 h.

Similarly, Jawaid et al. [66] reported that *S. aureus* growth was reduced after treatment with citronella essential oil nanoemulsion (from 6.0 to 3.9 log CFU/mL) and citronella essential oil (from 6.0 to 3.3 log CFU/mL), using MIC concentrations of the compounds, after 8 h of exposure. El-Sayed et al. [78] observed a 2.2 log cycle reduction in *S. aureus* count after 3 h of exposure to *T. vulgaris* nanoemulsion at 0.05% concentration, showing a bacteriostatic effect.

**Figure 3:**
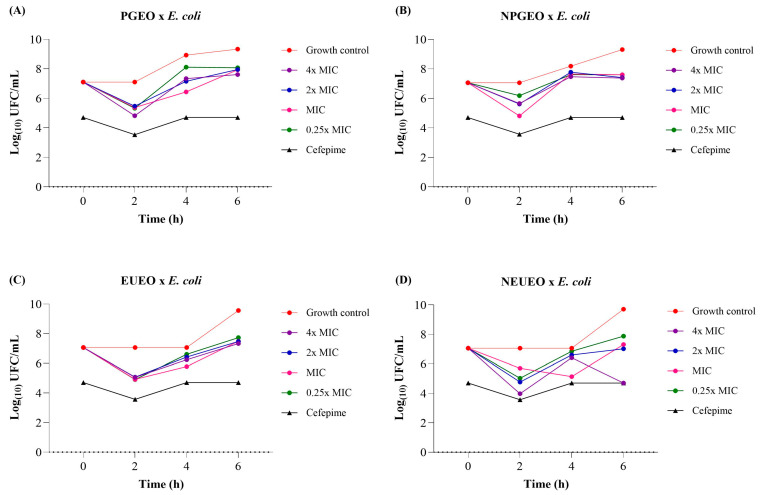
Time–growth inhibition assay of *E. coli* for different concentrations of (**A**) *P. guajava* essential oil (PGEO), (**B**) *P. guajava* nanoemulsion (NPGEO), (**C**) *E. uniflora* essential oil (EUEO), and (**D**) *E. uniflora* nanoemulsion (NEUEO). Based on the MIC determined for the essential oil and nanoemulsion, the tested concentrations were defined as 4 × MIC, 2 × MIC, MIC, and 0.25 × MIC. The control growth corresponds to positive microbial growth, and the MIC of cefepime was used as a growth inhibition control.

Figure 3 presents the time–growth inhibition assays of *E. uniflora* and *P. guajava* essential oils and nanoemulsions against *E. coli*, which had an initial inoculum size at time 0 of 7.06 log CFU/g. After 6 h of incubation, at all tested MIC concentrations, the *P. guajava* essential oil showed a count of 6.61–8.07 log CFU/g (Figure 3A), the *P. guajava* nanoemulsion showed a count of 7.38–7.60 log CFU/g (Figure 3B), the *E. uniflora* essential oil showed a count of 7.33–7.73 log CFU/g (Figure 3C), and the *E. uniflora* nanoemulsion showed a count of 4.70–7.88 log CFU/g (Figure 3D), often surpassing the initial inoculum value of 7.06 log CFU/g.

The positive microbial growth control presented values of 9.31–9.34 log CFU/g for the *P. guajava* essential oil and its nanoemulsion and 9.56–9.71 log CFU/g for the *E. uniflora* essential oil and its nanoemulsion after 6 h. The *E. uniflora* nanoemulsion, *P. guajava* essential oil, and *P. guajava* nanoemulsion tested at 4 × MIC demonstrated a reduction of 5.0, 2.7, and 2.2 log cycles, respectively, in the *E. coli* count compared to the positive microbial growth control. The other compounds showed a reduction of 1.3 to 1.8 log cycles in the *E. coli* count, indicating that the bacteriostatic activity against *E. coli* was dose-dependent.

The antibiotic cefepime showed a lower microbial count of 4.70 log CFU/g at 6 h compared to most tested compounds. Thus, it can be concluded that the tested compounds were more effective against *S. aureus* than against *E. coli*. El-Sayed et al. [78] observed similar results with the *T. vulgaris* nanoemulsion at a concentration of 0.05%, finding a lower reduction in viable *E. coli* count (0.86 log cycles) than *S. aureus* count, which demonstrated a 2.20 log cycle reduction after 3 h of exposure.

According to Chouhan et al. [72], Gram-positive bacteria are more susceptible to essential oils than Gram-negative bacteria are. This can be attributed to the fact that Gram-negative bacteria possess an outer membrane that is rich in lipopolysaccharides and more complex, limiting the diffusion of hydrophobic compounds through it. In contrast, this complex outer membrane is absent in Gram-positive bacteria, facilitating the access of hydrophobic compounds to the cell membrane. However, the bioactive components present in essential oils can bind to the cell surface and subsequently penetrate the phospholipid bilayer of the cell membrane in Gram-negative bacteria, leading to cell death.

In conclusion, the results of this study showed that, in the agar diffusion method, the *E. uniflora* nanoemulsion demonstrated the highest antimicrobial activity, followed by the *P. guajava* nanoemulsion, both of which outperformed their respective essential oils. The largest inhibition zones were observed against the yeasts *C. albicans* and *C. krusei* (in the case of the *E. uniflora* nanoemulsion), and inhibition zones were also noted for the Gram-positive bacterium *S. aureus* and the Gram-negative bacteria *P. aeruginosa*, *K. pneumoniae*, and *A. baumannii*. These findings highlight the importance of nanoemulsion formulation in enhancing the aqueous solubility of essential oils.

In the determination of MIC and MBC, the *E. uniflora* nanoemulsion showed the highest antimicrobial activity against the yeasts *C. albicans* and *C. krusei*, while the *P. guajava* nanoemulsion also exhibited low MIC and MBC values for *C. krusei*. However, for the tested bacteria, the essential oils exhibited lower MIC and MBC values than the nanoemulsions. In the microbial growth inhibition assay over time, both the nanoemulsions and essential oils displayed similar behavior, showing a better bacteriostatic effect against the Gram-positive bacterium *S. aureus* than against the Gram-negative bacterium *E. coli*.

Shahabi et al. [79] also reported that the antimicrobial effect of *Zataria multiflora* essential oil nanoemulsion strongly depended on the type of antimicrobial assay, the nanoemulsion concentration, the exposure time, and the type of bacteria tested.

## 3. Materials and Methods

### 3.1. Essential Oils of E. uniflora and P. guajava

Locally produced essential oils of *E. uniflora* and *P. guajava* with organic certification were purchased from a commercial establishment located in São Paulo, Brazil. Both essential oils were extracted from the leaves using a steam distillation technique according to the manufacturer protocols.

### 3.2. Identification of Major Volatile Compounds of E. uniflora and P. guajava Essential Oils

The essential oils of *E. uniflora* and *P. guajava* were subjected to chemical characterization using gas chromatography coupled with mass spectrometry (Agilent equipment, Model MSD5977B, Santa Clara, CA, USA). The chromatographic analysis parameters were as follows: injector temperature at 280 °C; injection volume: 1 μL; injection mode: split (1:20); flow rate: 1 mL min⁻^1^; carrier gas: helium; capillary column: DB-5MS (30 m × 0.25 mm × 0.25 μm); oven temperature gradient: initial temperature 60 °C, 2 min, rate of 4 °C/min up to 200 °C, and rate of 6 °C/min up to 260 °C, 10 min; mass detector temperature: 260 °C; ion source temperature: 280 °C; acquisition mode: scan. The compounds were identified by comparing the mass spectra of the peaks with those in the NIST17.L library (NIST Chemistry WebBook—webbook.nist.gov), and the similarity degree of each identification is presented in the results. The relative percentage area of each peak was calculated based on the sum of areas of all eluted peaks from the column and originating from the analyzed sample.

### 3.3. Determination of Total Phenolic Compounds and Antioxidant Activity of E. uniflora and P. guajava Essential Oils

The total phenolic content was determined using the Folin–Ciocalteu method [80], and the results were expressed as mg gallic acid equivalent per mL (mg GAE/mL). The antioxidant activity was evaluated using the DPPH (2,2-diphenyl-1-picrylhydrazyl) free radical scavenging assay according to Rufino et al. [81] and ABTS (2,2-azinobis-3-ethylbenzthiazoline-6-sulphonic acid) free radical scavenging assay as described by Rufino et al. [82]. The results were expressed as µmol Trolox equivalents per mL of sample (µmol TE/mL). The Folin–Ciocalteu, DPPH, and ABTS reagents were from Sigma-Aldrich-Merck (Darmstadt, Germany).

### 3.4. Development of the P. guajava and E. uniflora Nanoemulsions

The nanoemulsions were prepared using *P. guajava* and *E. uniflora* essential oils at a proportion of 15% (*v*/*v*), Cremophor^®^ EL was obtained from Merck (Darmstadt, Germany) and used as a surfactant at a proportion of 30% (*v*/*v*), Plurol^®^ Oleique was provided by Gatefossè (Saint-Priest, France) and used as a co-surfactant at a proportion of 5% (*v*/*v*), and phosphate buffer was used at pH 5.5 at a proportion of 50% (*v*/*v*). A 15% (*v*/*v*) concentration of the essential oils of *E. uniflora* and *P. guajava* was used for the formulation of the nanoemulsions, as it was observed that for both essential oils, this concentration was above the minimum bactericidal concentration for all tested microorganisms. The surfactants were selected according to Cardoso et al. [83], who used Cremophor^®^ EL as a surfactant due to its non-irritant characteristics, combined with Plurol^®^ Oleique, a co-surfactant that reduces the surface tension of the nanoemulsion. The mixtures were homogenized using an ultrasonic processor (Vibra-Cell VC 750, Sonics & Materials Inc., Newtown, CT, USA). The formulations were sonicated for 20 min at an amplitude of 20%; each cycle consisted of a 30 s pulse on and a 30 s pulse off. During the process, the temperature was controlled using ice around the beaker. Finally, the nanoemulsions were centrifuged at 4000 rotations per minute (rpm) for 10 min to ensure that no phase separation occurred.

### 3.5. Characterization of E. uniflora and P. guajava Nanoemulsions

The droplet size, polydispersity index (PDI), and zeta potential of the *P. guajava* and *E. uniflora* nanoemulsions (diluted at a ratio of 1:50 in ultrapure water) were analyzed using a Zetasizer Nano ZS (Malvern, Worcestershire, UK). The droplet size was determined using the dynamic light scattering (DLS) technique, and the zeta potential was measured by electrophoretic mobility. The pH was measured with a digital pH meter (Digimed, model DM-22, São Paulo, Brazil) by directly immersing the electrode in the undiluted nanoemulsion at 25 ± 1 °C.

### 3.6. Stability Study of P. guajava and E. uniflora Nanoemulsions

A stability study of *P. guajava* and *E. uniflora* nanoemulsions was carried out for 30 days, with the samples stored in hermetically sealed 15 mL Falcon tubes, under three conditions: room temperature (25 ± 2 °C), refrigerated (4 ± 2 °C), and 40 ± 2 °C. At time intervals of 0, 1, 3, 7, 14, and 30 days of storage, the samples were tested for droplet size, PDI, zeta potential, and pH [84].

### 3.7. Study of the Antimicrobial Activity of Essential Oils and Nanoemulsions of P. guajava and E. uniflora

#### 3.7.1. Microorganisms and Inoculum Preparation

For the determination of antimicrobial activity of the essential oils of *E. uniflora* and *P. guajava* and the nanoemulsions, the following microorganisms were used: Gram-positive bacteria (*Staphylococcus aureus* ATCC 29213), Gram-negative bacteria (*Pseudomonas aeruginosa* ATCC 27853, *Klebsiella pneumoniae* ATCC BAA-1706, *Acinetobacter baumannii* ATCC 19606, and *Escherichia coli* ATCC 25922), and yeasts (*Candida albicans* ATCC 10231 and *Candida krusei* ATCC 6258). The bacterial inoculum was prepared through direct suspension of microbial growth in Mueller–Hinton broth (Himedia, Thane, India), with turbidity adjusted between 0.08 and 0.10 on a spectrophotometer (Hitach U-3900H, Tokyo, Japan) at 625 nm (equivalent to the McFarland standard 0.5 and 1.0 × 10^8^ CFU/mL) [85]. For the yeasts, the inoculum was prepared by direct suspension of microbial growth in RPMI broth (Merck, Darmstadt, Germany), and the inoculum was adjusted using a spectrophotometer (Hitach U-3900H, Tokyo, Japan) to an absorbance between 0.08 and 0.13 at a wavelength of 530 nm [86].

#### 3.7.2. Agar-Well Diffusion Assay

For the comparison of antimicrobial activity between the nanoemulsions and essential oils, the agar-well diffusion method was used, as the paper disk method was not suitable for the diffusion of the nanoemulsions in gel. Additionally, the essential oils were tested pure and diluted to 15% (*v*/*v*) using Mueller–Hinton broth and 4% Tween 80 (Sigma-Aldrich-Merck, Darmstadt, Germany), allowing for comparison with the nanoemulsions, which were formulated with a 15% concentration of essential oil. Thus, the microbial inoculum was seeded, with the aid of a sterile swab, on the surface of a Mueller–Hinton agar plate (Himedia, Thane, India) until a uniform smear was obtained. After the inoculum dried, 6 mm diameter wells were punched into the plates using a sterile stainless-steel puncher. Following that, 50 µL of each sample was added to the wells. The plates were incubated at 37 °C for 24 h. At the end of the incubation, the inhibition zones that had formed around the wells were measured in millimeters [87].

#### 3.7.3. Determination of Minimal Inhibitory Concentration (MIC) and Minimal Bactericidal Concentration (MBC)

The minimum inhibitory concentration (MIC) and minimum bactericidal concentration (MBC) were determined according to the methodology of the Clinical and Laboratories Standards Institute [85,86]. The microbial inoculum (1.0 × 10^8^ CFU/mL) was diluted in Mueller–Hinton broth (bacterial inoculum) or RPMI broth (yeast inoculum) at a ratio of 1:150, resulting in a concentration of 1.0 × 10^6^ CFU/mL. Then, the essential oil and nanoemulsion samples were diluted at different concentrations (MIC 0.20–25.00 mg/mL and MBC 2.00–55.00 mg/mL) in Mueller–Hinton broth (bacterial inoculum) or RPMI broth (yeast inoculum) using serial dilution. The assays were carried out in 96-well flat-bottom microplates in triplicate. Each well received 100 µL of the diluted sample and 100 µL of the microbial inoculum, resulting in a final inoculum concentration of 1.0 × 10^5^ CFU/mL in a total volume of 200 µL per well. For the positive microbial growth control, each well received 100 µL of broth and 100 µL of microbial inoculum. For the non-growth microbial control, 100 µL of Mueller–Hinton broth (bacterial inoculum) or RPMI broth (yeast inoculum) and 100 µL of the essential oils or nanoemulsions was used. To avoid interference from the coloration of the samples and the broth, 100 µL of Mueller–Hinton or RPMI broth and 100 µL of the essential oils or nanoemulsions were used as blanks. The microplates were incubated at 37 °C for 24 h for bacterial tests and 48 h for yeast tests. Then, the turbidity of each well was read at 620 nm using a Multiskan^®^ microplate reader (Thermo Fisher Scientific Inc., Waltham, Massachusetts, USA). For the confirmation of the MBC, 100 µL of each sample was seeded on Mueller–Hinton agar plates for 24 h (for bacteria) and on potato dextrose agar (Himedia, Thane, India) for 48 h (for yeasts) at 37 °C. MBC was determined as the lowest concentration that allowed no visible microbial growth in agar. For the confirmation of MIC, 20 µL of resazurin dye (Sigma-Aldrich-Merck, Darmstadt, Germany) at 0.01% (*w*/*v*) was added to each well containing 100 µL of each sample. Resazurin is a redox indicator used to assess cell viability. Initially non-fluorescent and blue, it turns pink and fluorescent when reduced to resorufin by oxidoreductases within viable cells [88]. The MIC was defined as the lowest concentration at which there was no change in color.

### 3.8. Time–Kill Kinetics Assays of E. uniflora and P. guajava Essential Oils and Nanoemulsions

Time–kill kinetics or time–growth inhibition assays of *E. uniflora* and *P. guajava* essential oils and nanoemulsions were conducted against *S. aureus* and *E. coli*. The bacterial inoculum (1.0 × 10^8^ CFU/mL) was diluted in Mueller–Hinton broth, resulting in a concentration of 1.0 × 10^6^ CFU/mL. The essential oils and nanoemulsions were tested at concentrations of at 4 × MIC, 2 × MIC, MIC, 0.5 × MIC, and 0.25 × MIC. The assays were carried out in 96-well flat-bottom microplates. Each well received 100 µL of the diluted sample and 100 µL of the microbial inoculum, resulting in a final inoculum concentration of 1.0 × 10^5^ CFU/mL in a total volume of 200 µL per well. For the positive microbial growth control, each well received 100 µL of broth and 100 µL of bacterial inoculum. The MIC of oxacillin was used as a growth inhibition control for *S. aureus*, and the MIC of cefepime was used as a growth inhibition control for *E. coli*. Samples were collected at 0, 2, 4, and 6 h and spread on Mueller–Hinton agar plates, which were then incubated for 24 h at 37 °C. The number of resulting colony-forming units in log10 (log10 CFU/mL) was plotted against time [89].

### 3.9. Statistics

All analyses were performed in triplicate, and the results are expressed as the mean ± standard deviation (SD). All data were treated with the aid of the STATISTICA software version 10.0. Analysis of variance (ANOVA) was performed to detect significant differences between the analyses, and when differences were statistically significant, Tukey’s test for mean comparisons was used.

## 4. Conclusions

The results of this study demonstrated the promising potential of using *E. uniflora* and *P. guajava* essential oils for the formulation of gel-based nanoemulsions, which exhibited droplet sizes below 200 nm and stability for 30 days under ambient storage conditions. The *E. uniflora* nanoemulsion showed promising antifungal activity against the yeasts *C. albicans* and *C. krusei*, while the *P. guajava* nanoemulsion also exhibited low MIC and MBC values against *C. krusei*. The nanoemulsions developed in this study also demonstrated sufficient inhibitory effects against the tested Gram-negative and Gram-positive bacteria. Further studies are needed to evaluate the cytotoxicity and biocompatibility of gel-based nanoemulsions of *E. uniflora* and *P. guajava* to verify the safety of these formulations for human or animal cells. Therefore, the gel-based nanoemulsions of *E. uniflora* and *P. guajava* can be used as suitable antimicrobial compounds against pathogenic yeasts and bacteria. Future studies will focus on the development of dressings with bacteriostatic effects to aid in the treatment of wounds and burns.

## Figures and Tables

**Table 1:** Chemical composition (%) of *E. uniflora* essential oil.

	Components	Composition (%)	Classification
1	β-Myrcene	0.63	Monoterpene
2	α-Felandrene	0.17	Monoterpene
3	β-Thujene	0.20	Monoterpene
4	Trans-β-Ocimene	1.30	Monoterpene
5	β-Ocimene	3.32	Monoterpene
6	γ-Terpinene	0.15	Monoterpene
7	Terpinolene	0.14	Monoterpene
8	δ-Elemene	1.20	Sesquiterpene
9	α-Cubebene	0.31	Sesquiterpene
10	Copaene	0.30	Sesquiterpene
11	β-Elemene	4.74	Sesquiterpene
12	β-Caryophyllene	5.16	Sesquiterpene
13	γ-Elemene	1.72	Sesquiterpene
14	Bicyclosesquifelandrene	0.16	Sesquiterpene
15	Humulene	0.43	Sesquiterpene
16	Alloaromadendrene	0.53	Sesquiterpene
17	β-Panasinsene	0.71	Sesquiterpene
18	β-Cubebene	6.79	Sesquiterpene
19	β-Selinene	0.47	Sesquiterpene
20	Curzerene	34.80	Sesquiterpene
21	δ-Guaiene	1.34	Sesquiterpene
22	γ-Cadinene	0.14	Sesquiterpene
23	δ-Cadinene	2.01	Sesquiterpene
24	α-Cadinene	0.40	Sesquiterpene
25	Germacrene B	11.92	Sesquiterpene
26	Spathulenol	1.14	Sesquiterpene
27	Globulol	0.80	Sesquiterpene
28	Aristoladiene	3.28	Sesquiterpene
29	Germacrone	3.99	Sesquiterpene
30	Calamenene	4.62	Sesquiterpene
31	Aristolene	5.73	Sesquiterpene
	Monoterpenes		5.91%
	Sesquiterpenes		92.69%
	% of Identification		98.60%

**Table 2:** Chemical composition (%) of *P. guajava* essential oil.

	Components	Composition (%)	Classification
1	α-Pinene	0.24	Monoterpene
2	p-Cimene	0.18	Monoterpene
3	Limonene	0.75	Monoterpene
4	Eucalyptol	4.58	Monoterpene
5	α-Terpineol	0.19	Monoterpene
6	Copaene	0.80	Sesquiterpene
7	β-Caryophyllene	25.92	Sesquiterpene
8	Humulene	3.95	Sesquiterpene
9	β-Panasinsene	2.17	Sesquiterpene
10	β-Selinene	22.64	Sesquiterpene
11	γ-Selinene	19.13	Sesquiterpene
12	α-Muurolene	0.63	Sesquiterpene
13	β-Bisabolene	0.17	Sesquiterpene
14	γ-Cadinene	2.69	Sesquiterpene
15	7-epi-α-Selinene	0.49	Sesquiterpene
16	δ-Cadinene	2.48	Sesquiterpene
17	α-Calacorene	0.23	Sesquiterpene
18	Caryophyllene Oxide	5.61	Sesquiterpene
19	Humulene Oxide	0.37	Sesquiterpene
20	t-Cadinol	1.34	Sesquiterpene
21	Selin-6-en-4-α-ol	3.54	Sesquiterpene
	Monoterpenes		5.94%
	Sesquiterpenes		92.16%
	% of Identification		98.10%

**Table 3:** Antioxidant activity and content of total phenolic compounds of essential oils from *E. uniflora* and *P. guajava.*

Analyses	Essential Oils	
	*E. uniflora*	*P. guajava*
ABTS (μmol TE/mL)	853.62 ± 29.74 ^a^	204.19 ± 25.01 ^b^
DPPH (μmol TE/mL)	183.71 ± 8.43 ^a^	51.30 ± 20.01 ^b^
Phenolic Compounds (mg GAE/mL)	1.30 ± 0.35 ^a^	1.11 ± 0.64 ^b^

Values represent mean ± SD. Different letters within the same line mean significant differences at *p* < 0.05 according to the Tukey test at the 95% confidence level.

**Table 4:** Characterization of nanoemulsions of *E. uniflora* and *P. guajava.*

Analyses	Nanoemulsions	
	*E. uniflora*	*P. guajava*
Size (nm)	105.30 ± 0.60 ^a^	99.50 ± 0.70 ^b^
PDI	0.32 ± 0.01 ^b^	0.43 ± 0.01 ^a^
Zeta Potential (mV)	−9.29 ± 0.20 ^a^	−7.34 ± 0.20 ^b^
pH	5.75 ± 0.01 ^a^	5.41 ± 0.10 ^a^

PDI: polydispersity index. Values represent mean ± SD. Different letters within the same line mean significant differences at *p* < 0.05 according to the Tukey test at the 95% confidence level.

**Table 5:** Antimicrobial activity of *E. uniflora* and *P. guajava* essential oils and its nanoemulsions using agar-well diffusion method.

Microorganisms	Zone Diameter (mm)					
	EUEO (Pure)	EUEO (15%)	NEUEO	PGEO (Pure)	PGEO (15%)	NPGEO
*S. aureus*	8.04 ± 0.25 ^c^	9.62 ± 0.34 ^b^	8.41 ± 0.96 ^c^	9.10 ± 0.19 ^b^	10.93 ± 0.60 ^a^	8.02 ± 0.33 ^c^
*E. coli*	n	n	n	n	n	n
*K. pneumoniae*	n	n	15.13 ± 0.45 ^a^	n	n	9.97 ± 0.75 ^b^
*P. aeruginosa*	n	n	9.31 ± 0.39 ^a^	n	n	10.05 ± 0.39 ^a^
*A. baumannii*	n	n	10.79 ± 0.34 ^a^	n	n	6.06 ± 4.04 ^b^
*C. albicans*	7.78 ± 0.32 ^c^	14.30 ± 9.53 ^b^	20.97 ± 3.09 ^a^	n	n	n
*C. krusei*	6.67 ± 4.45 ^c^	9.40 ± 0.40 ^b^	15.20 ± 0.85 ^a^	n	n	n

EUEO = *E. uniflora* essential oil; NEUEO = nanoemulsion of *E. uniflora* essential oil; PGEO = *P. guajava* essential oil; NPGEO = nanoemulsion of *P. guajava* essential oil; n = no inhibition zone. Values represent mean ± SD. Different letters within the same line mean significant differences at *p* < 0.05 according to the Tukey test at the 95% confidence level.

**Table 6:** Minimum inhibition concentration (MIC) and minimum bactericidal concentration (MBC) of *E. uniflora* and *P. guajava* essential oils and their nanoemulsions.

Microorganisms	EUEO		NEUEO		PGEO		NPGEO	
	(mg/mL)							
	MIC	MBC	MIC	MBC	MIC	MBC	MIC	MBC
*S. aureus*	1.95 ^e^	17.53 ^b^	3.38 ^d^	30.46 ^a^	1.13 ^f^	10.16 ^c^	11.34 ^c^	>55.00
*E. coli*	5.84 ^d^	52.59 ^a^	11.28 ^c^	>55.00	0.42 ^f^	3.86 ^e^	3.52 ^e^	31.64 ^b^
*K. pneumoniae*	1.39 ^f^	12.49 ^c^	8.57 ^d^	>55.00	5.65 ^e^	50.81 ^a^	23.50 ^b^	>55.00
*A. baumannii*	0.25 ^g^	2.34 ^e^	6.31 ^d^	54.81 ^a^	1.22 ^f^	11.01 ^b^	5.95 ^c^	53.57 ^a^
*P. aeruginosa*	5.75 ^e^	51.71 ^b^	20.08 ^c^	>55.00	4.61 ^f^	41.49 ^a^	8.58 ^d^	>55.00
*C. albicans*	8.50 ^d^	>55.00	1.22 ^f^	11.02 ^b^	5.74 ^e^	51.66 a	9.07 ^c^	>55.00
*C. krusei*	4.58 ^d^	41.19 ^a^	0.54 ^g^	4.84 ^d^	3.48 ^e^	31.33 ^b^	1.26 ^f^	11.35 ^c^

EUEO = *E. uniflora* essential oil; NEUEO = nanoemulsion of *E. uniflora* essential oil; PGEO = *P. guajava* essential oil; NPGEO = nanoemulsion of *P. guajava* essential oil; Different letters within the same line mean significant differences at *p* < 0.05 according to the Tukey test at the 95% confidence level.

## Data Availability

The data presented in this study are available on request from the corresponding author. The data are not publicly available due to privacy interests.
